# Characterization of Genetic Variants of Uncertain Significance for the *ALPL* Gene in Patients With Adult Hypophosphatasia

**DOI:** 10.3389/fendo.2022.863940

**Published:** 2022-04-14

**Authors:** Raquel Sanabria-de la Torre, Luis Martínez-Heredia, Sheila González-Salvatierra, Francisco Andújar-Vera, Iván Iglesias-Baena, Juan Miguel Villa-Suárez, Victoria Contreras-Bolívar, Mario Corbacho-Soto, Gonzalo Martínez-Navajas, Pedro J. Real, Cristina García-Fontana, Manuel Muñoz-Torres, Beatriz García-Fontana

**Affiliations:** ^1^ Department of Medicine, University of Granada, Granada, Spain; ^2^ Instituto de Investigación Biosanitaria de Granada, Granada, Spain; ^3^ Department of Computer Science and Artificial Intelligence, University of Granada, Granada, Spain; ^4^ Andalusian Research Institute in Data Science and Computational Intelligence (DaSCI Institute), Granada, Spain; ^5^ European University Miguel de Cervantes, Valladolid, Spain; ^6^ Clinical Analysis Unit, University Hospital Clínico San Cecilio, Granada, Spain; ^7^ Endocrinology and Nutrition Unit, University Hospital Clínico San Cecilio, Granada, Spain; ^8^ Gene Regulation, Stem Cells and Development Lab, Pfizer-University of Granada-Junta de Andalucía Centre for Genomics and Oncological Research (GENYO), Granada, Spain; ^9^ Department of Biochemistry and Molecular Biology I, Faculty of Science, University of Granada, Granada, Spain; ^10^ Biomedical Research Network in Fragility and Healthy Aging (CIBERFES), Instituto de Salud Carlos III, Madrid, Spain

**Keywords:** alkaline phosphatase, bone, hypophosphatasia, mineralization, genetic variant, enzymatic activity, pyridoxal 5´ phosphate

## Abstract

Hypophosphatasia (HPP) a rare disease caused by mutations in the *ALPL* gene encoding for the tissue-nonspecific alkaline phosphatase protein (TNSALP), has been identified as a potentially under-diagnosed condition worldwide which may have higher prevalence than currently established. This is largely due to the overlapping of its symptomatology with that of other more frequent pathologies. Although HPP is usually associated with deficient bone mineralization, the high genetic variability of *ALPL* results in high clinical heterogeneity, which makes it difficult to establish a specific HPP symptomatology. In the present study, three variants of *ALPL* gene with uncertain significance and no previously described (p.Del Glu23_Lys24, p.Pro292Leu and p.His379Asn) were identified in heterozygosis in patients diagnosed with HPP. These variants were characterized at phenotypic, functional and structural levels. All genetic variants showed significantly lower *in vitro* ALP activity than the wild-type (WT) genotype (*p*-value <0.001). Structurally, p.His379Asn variant resulted in the loss of two Zn^2+^ binding sites in the protein dimer which may greatly affect ALP activity. In summary, we identified three novel *ALPL* gene mutations associated with adult HPP. The correct identification and characterization of new variants and the subsequent study of their phenotype will allow the establishment of genotype-phenotype relationships that facilitate the management of the disease as well as making it possible to individualize treatment for each specific patient. This would allow the therapeutic approach to HPP to be personalized according to the unique genetic characteristics and clinical manifestations of each patient.

## 1 Introduction

Hypophosphatasia (HPP) is a genetic disease characterized mainly by a lack of bone and tooth mineralization (1). In 2011 the prevalence of HPP in Europe was estimated at 1/300,000 for severe forms and 1/6,370 for moderate forms (2). In 2019, Garcia-Fontana et al. showed that, the estimated prevalence of mild HPP could be double that of previously reported estimation for Spanish population (1/3,100 vs. 1/6,370), and there could be more than 15,000 potential patients affected with mild forms of HPP underdiagnosed due to a lack of recognition in clinical practice (3). The clinical manifestations and prevalence of HPP has changed significantly between 1997 and 2019 from a rare and mostly recessive bone disease to a multisystemic, mostly dominant and frequent disease in its moderate and mild form (4).

In humans, the *ALPI, ALPP, ALPPL2* and *ALPL* genes encode intestinal (IALP), placental (PALP), tissue-specific germ cell (GCALP) and non-tissue-specific (TNSALP) ALP isoenzymes, respectively (4). The role of ALP is to hydrolyze phosphomonoesters with the release of inorganic phosphate (5). HPP is caused by the presence of loss-of-function mutations in the *ALPL* gene, resulting in decreased enzyme activity and consequent accumulation of the natural substrates of the enzyme (6). The extracellular substrates of TNSALP are pyridoxal 5´ phosphate (PLP) and inorganic pyrophosphate (PPi) (7, 8). Recent studies also suggest that adenosine triphosphate (ATP), di-phosphoryl lipopolysaccharide (LPS), and phosphorylated osteopontin (p-OPN) are also natural substrates of TNSALP (7, 9, 10).

**Figure 1:**
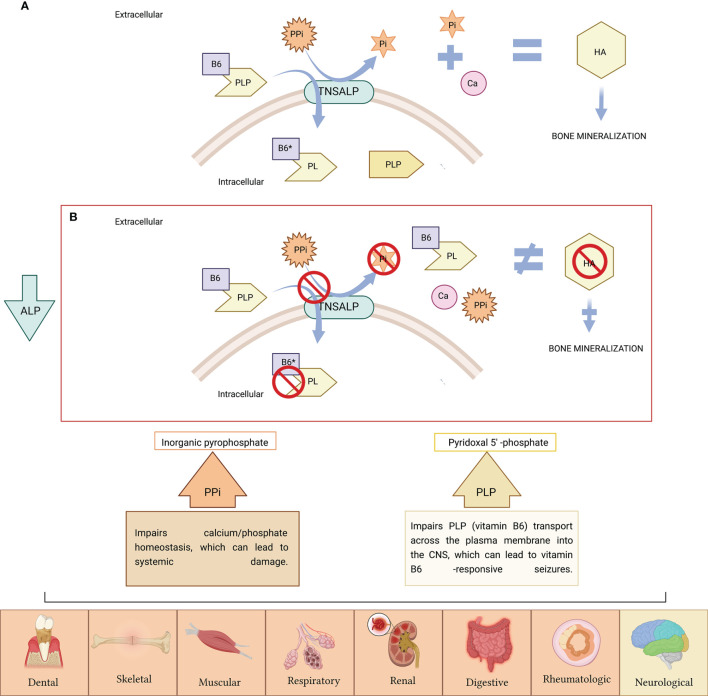
**(A)** TNSALP is attached to the cell membrane, where it degrades inorganic pyrophosphate (PPi) to inorganic phosphate (Pi), needed for hydroxyapatite (HA) crystal formation and further bone mineralization. TNSALP hydrolyses PLP (active form of B6) to pyridoxal (PL) in order to cross the cell membranes, after which is, then intracellularly rephosphorylated into PLP. **(B)** PPi is not degraded to Pi. Excess of PPi inhibits HA crystal formation with further inhibition of bone mineralization. PLP is not hydrolyzed to pyridoxal (PL), and accumulates extracellularly. The low activity of ALP causes the accumulation of PPi and PLP, causing symptoms in various organs of the human body (the symptoms related to PPi accumulation appears in orange boxes while symptoms related to PLP accumulation as vitamin B6 deficiency are indicated in yellow box). Figure created with BioRender.com.

The *ALPL* gene is ubiquitously expressed in the body participating TNSALP in numerous pleiotropic processes (11, 12). However, it is particularly expressed in bone, liver and kidney. The most frequent clinical manifestations of HPP are at the bone level. A hallmark of HPP is increased rates of fragility fractures and recurrent bone marrow injuries due to defective bone mineralization (13). In addition, generalized TNSALP deficiency is often associated with a rare form of rickets and osteomalacia (13–16). This does not apply to all patients due to the wide variety of genetic mutations and to the different forms of inheritance, which classifies HPP as a multisystemic disease due to its extraordinary heterogeneity. Phenotypes range from total absence of bone mineralization and fetal death mainly due to thoracic deformities and lung hypoplasia (17) to a wide variety of manifestations in different organs and systems including dental (13, 18–20), skeletal (13, 18–20), muscular (21), respiratory (22), neurological (23), renal (24) and rheumatological (25) and/or neurological symptoms (26) (**Figure 1**). However, some HPP patients seems to be asymptomatic, presenting non clinical manifestations often in adult HPP form.

According to the latest update of the Leiden Open Variation Database (LOVD) web site, about 500 loss-of-function variants of the *ALPL* gene have been described to date (https://databases.lovd.nl/shared/genes/ALPL) (6). The large variety of nonsense variants and the dominant negative effect of some variants contribute greatly to the clinical heterogeneity (6). This diverse clinical feature has led to the classification of HPP in different clinical forms from more severe to milder forms into perinatal lethal HPP, infantile HPP, childhood-onset HPP, adult HPP, odontohypophosphatasia and benign perinatal HPP (14, 17). In general, earlier onset subtypes are more severe and are inherited recessively, while moderate to mild late onset subtypes can be inherited in an autosomal recessive or dominant pattern (26).

The American College of Medical Genetics and Genomics (ACMG) together with the Association of Molecular Pathology and the College of American Pathologists have proposed new terminology in the interpretation of nucleotide changes occurring in genes for various pathologies (27). Pathogenic variant: variant that is certain to disrupt gene function or to be disease causing; probably pathogenic variant: the nucleotide change has a greater than 90% chance of being pathogenic; variant of uncertain significance (VUS): it is not possible to define whether it is pathogenic or not; variant probably non-pathogenic: the nucleotide change has a greater than 90% probability of not being pathogenic; non-pathogenic variant: a variant that has been shown to have no functional effect. Currently, there are computer tools as VarSome that classify variants in the criteria previously described (28).

Identifying and characterizing each genetic variant non previously described in order to determine whether particular variants give rise to particular phenotypes it would be interesting to know with greater certainty the possible effects it could have on the carrier patient. In this context, the aim of this study is to characterize at functional and structural level, three new VUS of the *ALPL* gene in HPP patients. In this way, genotype-phenotype relationships could be established for each patient. This would allow HPP therapy to be more targeted and personalized according to the genetic and clinical characteristics of each patient.

## 2 Material and Methods

### 2.1 Study Population

The subjects included were evaluated at the Clinical Analysis Unit of the University Hospital San Cecilio of Granada following the algorithm detailed by García-Fontana et al. (3). The clinical records of selected subjects were reviewed to exclude those subjects with low ALP levels caused by other causes different to HPP as certain therapies, malnutrition, zinc or magnesium deficiency, celiac disease among other, that could generate transiently or precipitously low ALP levels (29, 30). Subjects selected signed an informed consent and an individualized and personal interview about potentially related HPP symptoms was conducted. Two venous blood samples were collected from each subject at the Clinical Analysis Unit for PLP determination and for the genetic study. The present study was approved by the ethics committee of the University Hospital San Cecilio of Granada in accordance with the principles of the World Medical Association Declaration of Helsinki (Project ID: 0777-M1-20. Research Ethics Committee of Granada Center (CEI-Granada) on 8 May 2019).

### 2.2 Biochemical Analyses

#### 2.2.1 Alkaline Phosphatase (ALP)

ALP activity was measured in blood samples by absorption spectrophotometry on AU5800 analyzers (Beckman Coulter, California, USA) according to the method recommended for the “International Federation for Clinical Chemistry” (IFCC). ALP activity was determined by measuring the conversion rate of p-nitrophenyl phosphate (pNPP) to p-nitrophenol (pNP) in the presence of magnesium and zinc ions and of 2-amino-2-methyl-1-propanol (AMP) as a phosphate acceptor at pH 10.4. The rate of change in absorbance due to pNP formation was measured bichromatically at 410/480 nm Routine ALP determinations were performed at the Clinical Analysis Laboratory of the University Hospital Clínico San Cecilio of Granada. ALP activity reference values were 38-106,5 IU/L.

#### 2.2.2 Pyridoxal 5´ Phosphate

Plasma PLP concentrations, the active form of vitamin B6 and one of the main substrates of TNSALP, were determined by high-pressure liquid chromatography (HPLC) in the Clinical Analysis Unit of the University Hospital Niño Jesús (Madrid). Chromatographic determination was performed in an isocratic HPLC system with a fluorescence detector. The excitation and emission wavelengths were 320 nm and 415 nm, respectively. PLP reference values were 5 to 50 μg/L.

### 2.3 Sequencing of *ALPL* Gene

DNA extraction from peripheral blood lymphocytes was performed followed by amplification of the *ALPL* gene by polymerase chain reaction (PCR) according to the methodology described by Riancho et al. (31). Subsequent Sanger sequencing was performed using the PCR product, in order to know the sequence of the coding regions and exon-intron junctions of the *ALPL* gene, using the truncated sequence NM_000478.5 as a reference. The sequencing reaction product was read on an automated sequencer by capillary electrophoresis. Next, a copy number variant study was performed by multiple ligation probe amplification (MLPA) (MRCHolland) and subsequent analysis of results using the SeqPilot program (JSI Medical Systems). The results of the sequencing of the *ALPL* gene in peripheral blood DNA were provided by the Biomedical Diagnostic Center of the Clinic Hospital of Barcelona.

### 2.4 Cell Culture

In this study human embryonic kidney cell line (HEK293T) were cultured in 75cm^2^ filter cap cell culture flasks containing Dulbecco’s Modified Eagle Medium (DMEM) High Glucose with pH = 7.2 (Biowest), and 10% fetal bovine serum (FBS) (Capricorn scientific), 5% Ham’s F-12 (Biowest) and 1% of 100X antibiotic-antimicotic (Biowest). The cell culture flasks were maintained in an incubator at 37°C with 5% CO_2_ until the cells reached confluence of 70–80%. To suspend cells, 3mLof 10X Trypsin-EDTA solution (Sigma Aldrich) was used and the cells were successfully transferred to the 24-well cell culture plates. All cell culture plates were incubated at 37°C with 5% CO_2_.

### 2.5 Plasmids Design and Amplification

The vectors used throughout this study were constructed by modifying the plasmid pcDNA3.1. The *ALPL* gene with the different mutations under study (pcDNA3.1:*ALPL* c.Del69_74, pcDNA3.1:*ALPL* c.1135C>A and pcDNA3.1:*ALPL* c.875C>T) was inserted into this plasmid. The plasmid pcDNA3.1 with the *ALPL* wild-type (WT) gene insert (pcDNA3.1:*ALPL*) was used as a control to functionally characterize the identified variants. The plasmid without any insert (empty pcDNA3.1) was used as an internal control to check the basal expression of the *ALPL* gene at the cellular level. The *ALPL* gene sequences and their corresponding gene variants were each inserted independently into the multicloning site, with flanking sequences for the *HindIII* and *BamHI* enzymes. All vectors were supplied by GenScript.

To amplify the plasmid concentration, 1 μg of each plasmid vector was transferred separately to 50 μL of competent *E. coli* JMC109 strain by using heat shock method. Transformed *E. coli* cells were grown overnight on LB agar medium containing ampicillin at concentration of 100 mg/mL at 37°C. Then, all plasmid vectors were extracted by using kit NucleoBond^®^ Xtra Midi EF (Macherey-Nagel™) according to the manufacturers` instructions. The purity and concentration of the extracted plasmids was determined by spectrophotometric reading, using the NanoDrop Spectrophotometer (bioNovan). Samples with absorbance ratios 280/260 nm around 1.8 were selected. To ensure the complete integrity of the different plasmids, a PCR was performed confirming that the *ALPL* genes with the mutations and *ALPL* WT were correctly inserted into the plasmid. For this purpose, the primers *ALPL*-F: 5′-TGGCACCTGCCTTACTAACT-3′ and *ALPL*-R: 5′-CACGTTGGTGTTGAGCTTCT-3′ for plasmids containing some *ALPL* variant and the primers pCDNA3.1-F: 5’-CGTCACGCTGTAGGTATCTCAGTTC-3’ and pCDNA3. 1-R: 5’-GCCTACATACCTCGCTCTGCTAATC-3’ for empty pCDNA3.1 plasmid, were used. The PCR reaction was performed using the Horse-power Taq DNA Polymerase kit (Canvax) and following the protocol established by the manufacturer. Visualization of the process was performed by 2% agarose gel electrophoresis.

### 2.6 Transfection Studies

*ALPL* WT, *ALPL* mutants and pcDNA plasmids were transiently transfected into HEK293T cells for 48 hours. Transfections were performed by the lipofection method with LipoD293 DNA *In Vitro* Transfection Reagent (SignaGen Laboratories) following the manufacturer’s instructions. Specifically, 150,000 cells/well were seeded in 24-well plates and allowed to grow for 24 hours at 37°C and 5% CO_2_. For transfection, 1.5 μL of LipoD293, 500 ng of plasmid of interest and non-supplemented Advance DMEM were added to complete a final volume of 50 μL per well to be transfected.

### 2.7 Gene Expression Determination

RT-qPCR experiments were performed to control transfection and *ALPL* exogenous expression. The total RNA was isolated from each transfected culture using a *RNeasy* ^®^ Mini Kit (QIAGEN). RNA was treated with DNAse (Qiagen), then, cDNA was synthesized from 600 ng of total RNA using the iScript cDNA synthesis kit (BioRad) following the manufacturers’ instructions. Quantitative PCR was performed using the PowerUp SYBR Green Master Mix (Thermo Fisher Scientific) in a CFX96 Real Time thermocycler (BioRad). The set of primers designed to amplify a 121 bp fragment of the *ALPL* gene from both WT and mutant plasmids are the following: ALPL-F: 5′-TGGCACCTGCCTTACTAACT-3′ and ALPL-R: 5′-CACGTTGGTGTTGAGCTTCT-3′. Gene expression data were normalized to the expression of the reference gene Ribosomal Protein L13 (RPL13) and reported as normalized *ALPL* expression. The following set of primers was used to amplify the reference gene: RPL13-F: 5′-CGTAAGATCCGCAGACGTAAGGC-3′ and RPL13-R: 5′-GGACTTGTTCCGCCTCCTCGGAT-3′.A standard line was run at known cDNA concentrations (ng/μL) to determine the total amount of cDNA in our samples.

### 2.8 ALP Activity in Transfected Cells

The ALP activity was determined in triplicate using Alkaline Phosphatase Detection Kit (Abnova) following the methodology recommended by the manufacturer. The enzyme activity of ALP was measured with a spectrophotometer (Dynex Technologies) at 450 nm to detect the chromogenic product as a result of the ALP activity.

### 2.9 Cell Viability

The cell viability was analyzed in triplicate using FITC Annexin V Apoptosis Detection Kit I (BD Biosciences) following the steps indicated by the manufacturer. Viable cells are FITC Annexin V and propidium iodide (PI) negative and death cells are both FITC Annexin V and PI positive. Subsequently, a reading was performed on the BD FACSAriaTM II Cell Sorter flow cytometer.

### 2.10 Three-Dimensional (3D) Structural Modeling

To check the effect of the new mutations identified in this study, the 3D modeling of WT and TNSALP mutants was completed using SWISS MODEL (https://swissmodel.expasy.org/). The structure modeling is based on the sequence homology between TNSALP and the PALP (PDB ID: 1EW2)determined at a resolution of 1.8 Å (7, 32).The amino acid sequence of the human TNSALP molecule is 57% identical and 74% homologous with the human PLAP molecule (33). The new pdb files for WT, p.Del Glu23_Lys24, p.Pro292Leu, and p.His379Asn TNSALP are available upon request. Ribbon representations and hydrophobic surface representations were obtained using UCSF Chimera software. Mutation-related residues in the present study were positioned using the open source https://swissmodel.expasy.org/repository/uniprot//P05186.35.

### 2.11 Statistical Analyses

The ALP activity measure was calculated based on absorbance at 450 nm in three independent experiments in transfected cells, and Shapiro-Wilk test was used to study the distribution of this variable. ANOVA was performed to compare the differences between groups. Statistical significance was set at *p* < 0.05 (two-tailed). Statistical analysis was performed using SPSS version 22.0.

## 3 Results

### 3.1 Biochemical and Genetic Results

#### 3.1.1 Biochemical Analysis of ALP and PLP Levels in Serum Samples

ALP and PLP levels were analyzed from serum samples of subjects with suspected diagnosis of HPP due to low levels of ALP following the protocol described by García-Fontana et al. (3). Blood samples were sequenced in order to identify genetic variants of *ALPL* gene. The results of ALP and PLP values of the patients in whose a genetic variant of the *ALPL* gene was identified are summarized in **Table 1**.

**Table 1:** Biochemical determinations of ALP and PLP associated with mutations of uncertain significance.

Samples	c.Del69_74/p.Del Glu23_Lys24	c.875C>T/p.Pro292Leu	c.1135C>A/p.His379Asn	Reference range
ALP activity (IU/L)	23	16	10	38-106,5
PLP (μg/L)	34	119	462	5-50

The reference values were extrapolated from the values defined by the Clinical Analysis Unit of the University Hospital Niño Jesús (Madrid).

The p.Del Glu23_Lys 24 variant resulted in ALP activity values close to the lower limit of the normal range, while PLP levels were within the normal range (34μg/L). On the other hand, p.Pro292Leu and p.His379Asn variants showed abnormal levels of both ALP and PLP activity in blood. Particularly significant is the p.His379Asn variant, which has a very low ALP activity (10 IU) as well as drastically elevated levels of serum PLP (462 μg/L) (**Table 1**).

#### 3.1.2 Identification of Variants of Uncertain Significance

The mutations analyzed in this study were obtained from patients evaluated at the Clinical Analysis Unit of the University Hospital Clínico San Cecilio of Granada (Spain). Three mutations in the *ALPL* gene were identified by the sequencing service of the Center for Biomedical Diagnosis of the Hospital Clínic of Barcelona.

The 27-year-old patient presented a c.69_74del VUS in exon 3 and in heterozygosis, involving a deletion of glutamine 23 and lysine 24 in the TNSALP protein (p.Del Glu23_Lys24).

The 62-year-old patient presented the c.875C>T VUS in exon 9 and in heterozygosis, coding for the amino acid change from proline to leucine at position 292 of the TNSALP protein (p.Pro292Leu).

The 45-year-old patient presented a c.1135C>A VUS in exon 10 in heterozygosis, coding for the amino acid change from a histidine to an asparagine at position 379 of the TNSALP protein (p.His379Asn).

These three mutations were categorized as VUS by VarSome tool (https://varsome.com).

### 3.2 Clinical Manifestations

The symptomatology of these patients was highly heterogenous. Nevertheless, in all three cases, articular pain was observed, especially in the knees (**Table 2**).

**Table 2:** Clinical manifestations of patients affected with variants of uncertain significance of *ALPL* gene.

Samples	c.Del69_74/p.Del Glu23_Lys24	c.875C>T/p.Pro292Leu	c.1135C>A/p.His379Asn
Pathognomonic symptoms	Knee and lower back pain	Gonalgia	Episodes of paresthesia in face and upper limbs
		Meniscopathy	Elbow joint pain and fatigue
			Hip and lower limb pain
			Muscle weakness
Non-pathognomonic symptoms	Thyroid bulge	Benign prostatic hyperplasia	Drowsiness and tiredness
			Unfocused vision
			Achalasia and profuse salivation
			Kidney cramps
			Hypothyroidism

The most severe symptomatology is attributed to the p.His379Asn variant, while the clinical profiles derived to the p.Del Glu23_Lys24 and p.Pro292Leu variants were associated with a milder symptomatology. Interestingly, none of the 3 affected patients showed a clinical history of fractures, chondrocalcinosis or dentition abnormalities, symptoms characteristic of the moderate phenotypes associated with adult HPP, but presented with other complaints not associated, *a priori*, with HPP (**Table 2**).

According to the age of diagnosis of the different clinical manifestations in affected patients, the p.His379Asn variant has the earliest symptomatologic onset. Thus, the episodes of muscle weakness and pain in the upper and lower extremities began in childhood. From the age of 30 the patient presented episodes of extreme weakness and fatigue with inability to move the extremities and palpebral ptosis in addition to dysphonia related to exertion. At the age of 42 the patient suffered episodes of paresthesia in face and upper limbs as well as visual disturbances, kidney cramps, achalasia, profuse salivation and thyroid alterations (hypothyroidism). The patient was treated with meniston, corticoids, azathioprine, immunoglobulins and plasmapheresis without improvement. Additionally, the affected patient received treatment with carnicor, ubiquinol, bisoprolol, lexatin, melatonin, acfol, auxin A, collagen+Mg and probiotics with slight improvement. The current treatment includes eutirox only and has recently started with asfotase alfa therapy, which has resulted in a significant improvement, evidencing that most of the clinical manifestation of this patient may be related to HPP.

Regarding the p.Del Glu23_Lys24, the affected patient presented with knee and lumbar pain at the age of 28 years, without treatment. In addition, at the age of 23 she was diagnosed with bulge without thyroid hormone involvement and therefore did not require treatment.

The patient affected with p.Pro292Leu variant presented episodes of gonalgia, meniscopathy and benign prostatic hyperplasia at the age of 61 years, receiving treatment with topical anti-inflammatory drugs.

### 3.3 ALP Activity of Variants of TNSALP in An *In Vitro* Model

The ALP activity was measured in HEK293T cells transfected with plasmids containing the *ALPL* WT or the *ALPL* mutants. Empty pcDNA3.1 plasmid was used to measure basal ALP activity of the cells.

Cells containing *ALPL* WT had a statistically significant higher expression compared to the rest of the transfected cells. The p.Pro292Leu and p.His379Asn variants had no significant differences in the enzyme activity compared to TNSALP basal expression control.

**Figure 2:**
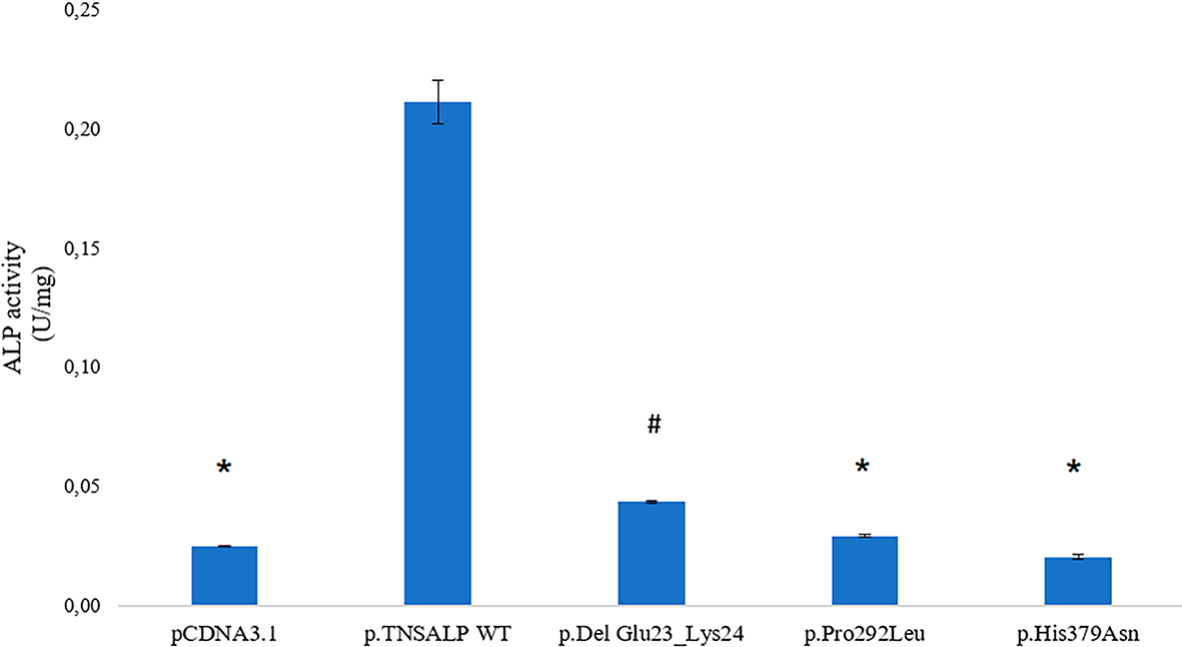
ALP activity in HEK293T cells transfected with pcDNA 3.1 or pcDNA 3.1+ insert. Quantitative results of the ALP assay expressed in U/mg are shown. Results are expressed as mean ± SEM of TNSALP activity. ANOVA was used for comparisons between groups. *p < 0.001 vs WT; ^#^ *p <* 0.001 vs all groups.

The p.Del Glu23_Lys24 variant showed significantly higher activity than the other variants and pcDNA3.1 control. The results are shown in **Figure 2**.

### 3.4 Absolute mRNA Expression of VUS of *ALPL* in an *In Vitro* Model

The determination of the absolute expression of VUS-associated mRNA was performed by qPCR of the different groups of HEK293T transfected with the corresponding plasmids.

**Figure 3:**
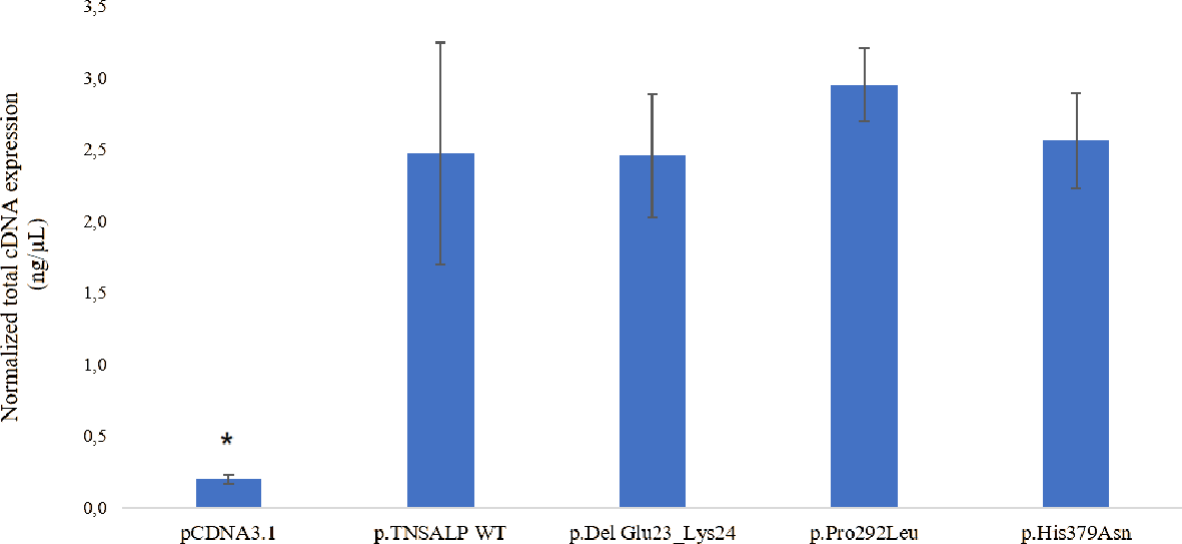
Total mRNA expression. The results are expressed as the percentage of expression of the Cts ± SEM normalized with the expression values of the constitutive gene RPL13. ANOVA corrected by Tukey’s test was used for comparisons between groups. to empty pcDNA3.1 are indicated with an *p < 0.05 vs all groups.

The transfection and expression studies performed to verify the validity of the experiment demonstrated that the *ALPL* transcriptional expression was similar in cells transfected with plasmids containing the *ALPL* WT or mutants (**Figure 3**). Statistically significant *ALPL* gene expression was found in *ALPL* WT cells or *ALPL* mutant cells compared to cells transfected with empty vector (**Figure 3**). The latter represents the endogenous expression of the *ALPL* gene in HEK293T cells (p-value<0.05).

### 3.5 Cell Viability Assay

**Figure 4:**
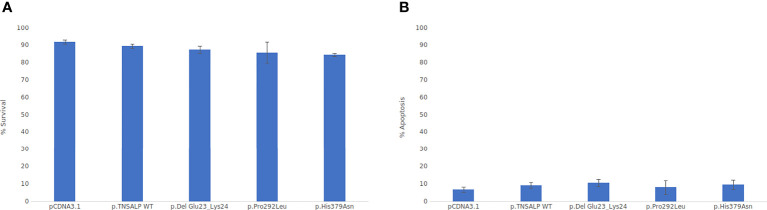
Viability cell assay graphs. ANOVA corrected by Tukey’s test was used for comparisons between groups Statistically significant differences was set at a *p-*value <0.05. **(A)** Percentage of cell viability. **(B)** Percentage of apoptotic cells.

Cell viability was assessed by flow cytometry. As depicted in **Figure 4**, there were no statistically significant differences in cell viability between the groups. All groups had a survival rate of around 85-90% (**Figure 4A**) and an apoptosis rate around of 5-10% (see **Figure 4B**).

### 3.6 Three-Dimensional Modelling of the Structure of the Variants of TNSALP

**Figure 5:**
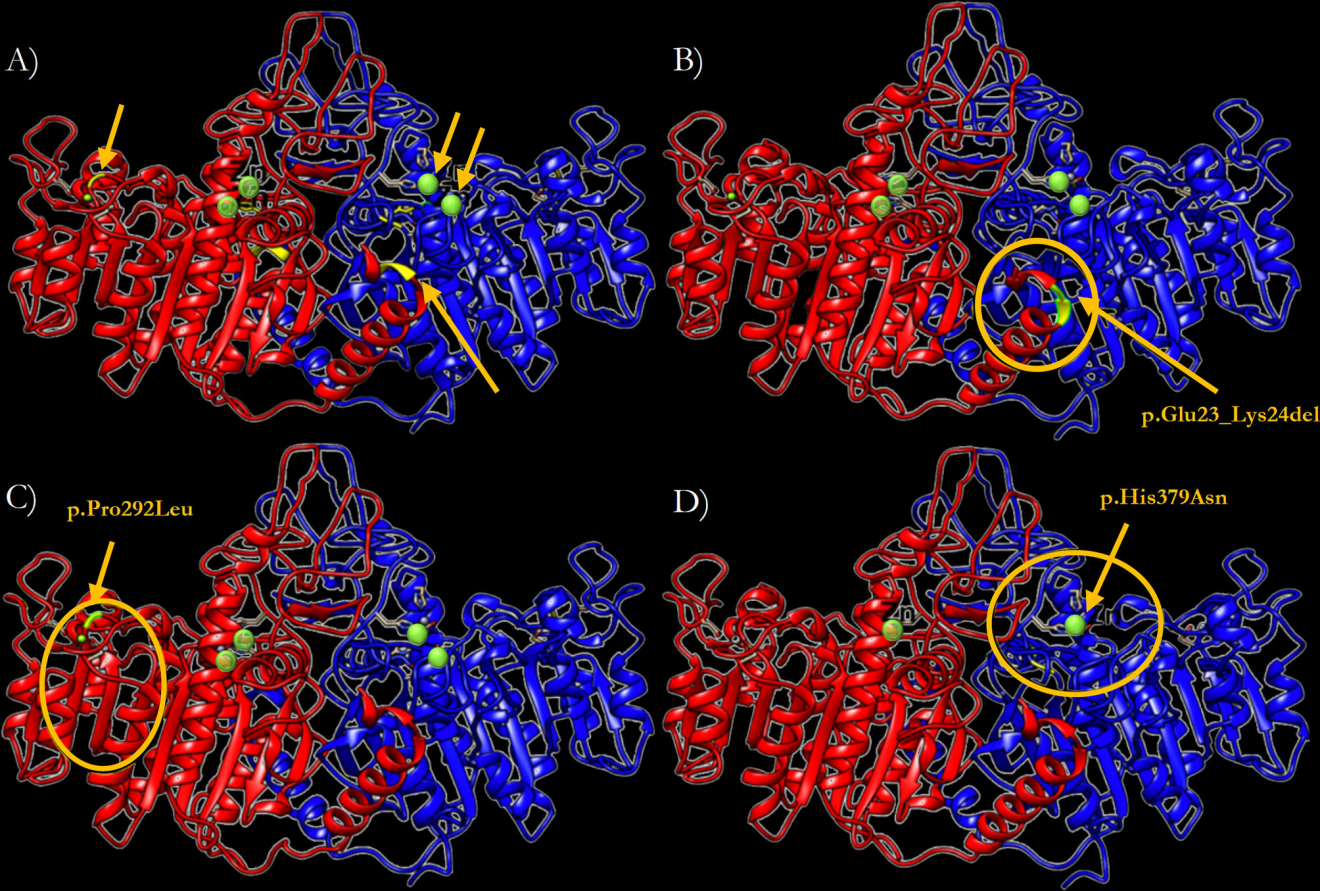
Three-dimensional representation of the TNSALP protein in the form of ribbons. **(A)** TNSALP WT. Zn^2+^ atoms bound to the corresponding Zn^2+^ binding sites are highlighted in green. The arrows indicate the location of the different mutations identified in this study. **(B)** TNSALP with the genetic variant p.Del Glu23_Lys24. The deletion is marked in yellow and the major structural changes are highlighted with a yellow circle. **(C)** TNSALP with the genetic variant p.Pro292Leu. The amino acid substitution is marked in yellow and the major structural changes are highlighted with a yellow circle. **(D)** TNSALP with the genetic variant p.His379Asn. The loss of 2 of the 4 Zn^2+^ binding sites in the dimeric protein is highlighted with a yellow circle in one of the protein monomers.

In order to predict the effect of the three newly identified mutations on the TNSALP structure, a 3D model based on the sequence homology between TNSALP and the PALP was obtained using the web-server SWISS MODE. **Figure 5A** shows the structure of the TNSALP WT protein. The yellow arrows and green dots indicate the locations of the mutations in the different variants described in this study

The p.Del Glu23_Lys24 variant showed significant changes at the end of the α-helix anchored to the N-terminal end, where the deletion occurs, leading to a shortening of the protein structure (**Figure 5B**). Secondly, the p.Pro292Leu variant showed an affectation of two of the β-strands that constitute the β-sheet, which acts as a structural domain and an integral part of the calcium-binding domain. This variant resulted in the elongation of these chains and reduction of the extension of the loops between them (**Figure 5C**). Finally, the p.His379Asn variant affected one of the two Zn^2+^ binding sites of each of the protein monomers. Although it seems to have no important structural impact, this change implies a reduction of the enzyme’s Zn^2+^ atom binding capacity by half to carry out its catalytic function (**Figure 5D**).

**Figure 6:**
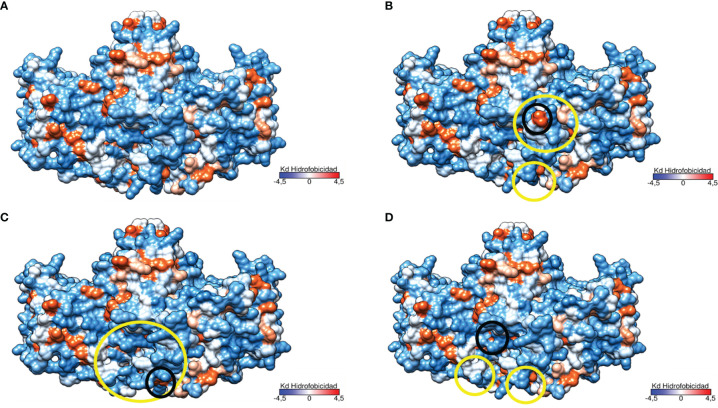
Three-dimensional representation of the TNSALP protein according to its hydrophobicity. Hydrophobicity is represented on the Kyte and Doolittle hydrophobicity scale (Kd scale); from the most hydrophilic amino acids in light blue to the most hydrophobic in red. Changes to more hydrophilic and hydrophobic amino acids are marked in yellow and black circles respectively. **(A)** TNSALP WT. **(B)** TNSALP with the genetic variant p.Del Glu23_Lys24. **(C)** TNSALP with the genetic variant p.Pro292Leu. **(D)** TNSALP with the genetic variant p.His379Asn.

The study of the protein polarity related to these genetic variants revealed significant changes in the hydrophobicity and folding in the area surrounding the mutation in the structure of the protein encoded by p.Del Glu23_Lys24 variant (**Figure 6**).This causes the exposure of two highly hydrophobic residues to the outside (see **Figure 6B**). The p.Pro292Leu variant caused polarity changes in the immediate vicinity of the mutation and at the homodimer interface leading to an increased gapping in the region as is represented in **Figure 6C**. Finally, the polarity changes related to the p.His379Asn variant were minimal, causing a little compaction of some exposed hydrophobic residues in the protein (**Figure 6D**). Notably, the three genetic variants resulted in a change in the lower region of the homodimer interface, affecting one of the characteristic clusters of hydrophilic residues present in TNSALP WT.

## 4 Discussion

In the present study we identified three uncertain variants into adult patients classified as VUS, as there was not yet sufficient evidence to categorize them according to its pathogenicity. However, the low serum levels observed in patients carrying these variants raise the suspicion that they could probably be pathogenic. Functional studies supported the results of serum determinations, showing significantly decreased enzyme activity in all three variants compared to TNSALP WT, showing the p.Del Glu23_Lys24 variant the higher enzymatic activity and the p.His379Asn variant the lower activity values. Regarding PLP levels, as expected, we found an inverse correlation with ALP activity, with gradually increasing serum PLP values associated with gradually decreasing ALP enzyme activity. Considering only the biochemical data, it seems difficult to establish a clear relationship between the severity of clinical manifestations and ALP enzyme activity, since of the two variants showing a major reduction in enzyme activity (p.Pro292Leu and p.His379Asn) only the latter is associated with a severe HPP phenotype. However, it appears that serum levels of PLP may better reflect the prognosis of the disease, and drastically elevated PLP levels may act as a marker of HPP severity.

Structural characterization allowed us to observe that p.Del Glu23_Lys24, the least severe variant at biochemical level, was strongly affected both structurally and in terms of polarity, particularly in the region of the N-terminal domain. The N-terminal domain region is composed of 22 residues with an important role in protein dimerization and is the anchoring site of glycosylphosphatidylinositol (GPI) to the TNSALP molecule (34). This deletion results in a shortening of the protein structure with a loss of the hydrophobic structure that protects the 22 residues of the N-terminal domain leading to the exposition of a hydrophilic residue to the surface which may affect the stability of the protein. The N-terminal domain of the monomeric subunits of TNSALP surrounds the active site of the enzyme (11). In fact, the correct folding of the N-terminal domain and the interaction with its microenvironment is crucial for both the structural integrity of the protein and for intramolecular transitions, so mutations in this area could lead to influence the allosteric performance of TNSALP as well as enzymatic catalysis of the active center (11, 35). Therefore, the p.Del Glu23_Lys24 mutation could compromise structural stability and alter interactions with the microenvironment leading to disturbed catalysis as our results showed. At clinical level, this variant seems to have a mild symptomatology although the early onset of the articular manifestations could imply a worsening of the symptomatology over time, so this patient should be followed up to study the phenotype evolution.

Although this variant was initially classified as VUS, based on our functional and structural characterization results as well as on the symptomatology of the carrier patient, we consider that the p.Del Glu23_Lys24 variant could be considered as likely pathogenic.

The structural change of TNSALP associated with p.Pro292Leu variant affects the structural domain involved in calcium binding. Although calcium binding is crucial for proper folding and assembly of the TNSALP molecule (15), and it appears to be fundamental to TNSALP activity in bone mineralization (36), the structural and functional significance of the calcium binding site in TNSALP is not yet entirely clear to date (37). Two variants at the same amino acid position have been described in compound heterozygosis in patients affected with HPP: [c.(98C>T); (874C>A) and c.(815G>T); (874C>T)] related to severe forms of HPP (infant HPP and lethal perinatal HPP, respectively) (38, 39).

On the other hand, the structural change in TNSALP derived from this variant mainly affect the β-strand generating the elongation of this chain which causes instabilities in the hydrophobic region. Other mutations previously described affecting parts of this β-strand (p.Val217Ala or p.Val217Lys) has been associated with perinatal lethal form of HPP (40), so in general, mutations in this strand of the protein seem to be associated to severe phenotypes.

The variant identified in our study (p.Pro292Leu), appears to be associated with a mild phenotype of HPP in contrast to the severe phenotypes associated with other mutations in the same region. This variant seems to be the least pathogenic of the three identified (at clinical level) since, in addition to presenting few symptoms related to HPP, the age of symptomatologic onset was quite late.

These results suggest that the severity of symptomatology appears to be closely related to the amino acid encoded at that position. In the variant identified in our study, the mild phenotype observed in the affected patient could be explained by the similarity in stereochemistry and charge of proline with leucine, leading to a less severe amino acid substitution. However, it must be considered that the severity of the other variants in the same position may be due to compound heterozygosity and different degrees of penetrance.

The p.His379Asn variant seems to be the one associated with a more severe form of HPP, and although at the structural level there are no significant changes in terms of structure or polarity, the amino acid change in the protein sequence seems to have an important functional repercussion. This variant results in an amino acid change that causes the loss of one of the Zn^2+^ binding sites of the protein. This cation located in the active site of TNSALP acts as a cofactor of the metalloenzyme. Therefore, the main cause of such a drastic decrease in enzyme activity seems to be associated with the 50% reduction in Zn^2+^ binding capacity. Accordingly, one of the characteristic symptoms of acrodermatitis enteropathica, a disease caused by a Zn^2+^ deficiency due to a mutation in SLC39A4 which encodes the zinc transporter Zip4, is hypophosphataemia (41). Based on this, we consider that this amino acid change close to the active center is particularly significant and helps to understand the severity of this variant. However, the heterozygous inheritance of this variant requires additional studies to rule out the presence of other mutations in non-coding regions of the *ALPL* gene that may explain the severity of the disease in the affected patient.

Despite the p.His379Asn variant has been associated to adult HPP the beginning of clinical manifestations in the affected patient was during childhood increasing the severity with age, ranging from fatigue and muscle pain in the lower limbs to hypotonia and paresthesias. This fact suggests that p.His379Asn variant could be associated with a form of infantile HPP, but due to the lack of knowledge of the HPP, its low prevalence and the absence of bone symptoms, there was a long delay in the diagnosis leading an accumulated damage until it was diagnosed as adult HPP (42).

Although some mutations have been identified in the other 5 residues that make up both Zn^2+^ binding sites (p.His341, p.His454, p.Asp337, p.Asp60, p.Asp378) that have been classified as potentially pathogenic, none of them had been characterized at the molecular level to date. However, the p.Asp378 variant has been associated with mild to severe phenotype of infantile HPP. Similarly, our findings supported by biochemical and clinical data as well as functional and structural characterization, reveal that p.His379Asn variant is clearly associated with a moderate to severe phenotype of HPP.

According to our results, of the three variants c.69_74del; p.(Del Glu23_Lys24), c.875C>T; p.(Pro292Leu) and c.1135C>A; p.(His379Asn) initially classified according to ACMG recommendations as VUS, the first has been reclassified as likely pathogenic and the two last as pathogenic in Varsome web search (https://varsome.com/) (28).

Interestingly, none of the patients had any fracture event in their clinical history. As mentioned above, this may be because TNSALP is not only involved in the mineralization process but also has pleiotropic functions in the body (11, 12).

Although this study is more oriented to find a genotype-phenotype relationship between HPP and mutations in the ALPL gene, it is important to point out that there are other less common situations that are also associated with low levels of ALP which must be considered to make a correct diagnosis. In this line, iron and ferritin has been shown as potent inhibitors of osteogenesis, significantly inhibiting ALP activity considering the ferroxidase activity of ferritin as the central element of this inhibition (43). In accordance with this, there are some studies that shows the involvement of other factors in TNSALP regulation such as the transcription factor RUNX2 (44). Similarly, it has been described other regulatory factors as PHOSPHO1, a factor responsible for generating Pi for HA crystallization with non-redundant role for TSNALP or ENPP1 that acts as a phosphatase in the absence of TNSALP (45). In this context, the existence of other modifier genes not yet known or identified cannot be excluded regarding the development of the heterogeneous clinical manifestations in the HPP patients.

In addition, the epigenetic modifications could contribute to the severity grade of the clinical behavior of the disease. In this line, the study of Delgado-Calle et al. shows an important role of DNA methylation in the regulation of ALPL expression through the osteoblast-osteocyte transition (46). Additionally, different lifestyles or behaviors should be considered as modifiers factors of HPP phenotype since seem to be a direct effect in ALP levels. Thus, the physical activity has been directly related to increased levels of ALP (47, 48). Regarding the study of phenotype associated to HPP, maybe it worthy to explore the role of these regulatory factors as well as the contribution of external factors as these lifestyles or behaviors.

This study highlights the importance of making a correct diagnosis of HPP in addition to establishing a geno-phenotypic relationship whenever possible. This will allow on the one hand to avoid erroneous or late diagnoses such as the case of the patient with p.His379Asn variant or erroneous treatments such as treatment with bisphosphonates, which worsen the symptoms derived from the hypomineralization of HPP by decreasing the activity of TNSALP (49); on the other hand, it will improve the knowledge of this metabolic disorder to make it accessible to the scientific community, allowing a better management of the disease through the establishment of a personalized medicine based on to the unique genetic characteristics and clinical manifestations of each patient.

## 5 Conclusions

Due to the high heterogeneity in the symptoms of HPP, it is necessary to characterize the new genetic variants that are identified in order to establish a genotype-phenotype relationship, which allows the most appropriate therapeutic measures to be carried out in each case. Functional and structural characterization studies showed that the p.Del Glu23_Lys24 and p.Pro292Leu variants are associated with mild adult HPP despite the marked reduction in enzymatic ALP activity. The variant p.Pro292Leu appears to be associated with the mildest symptomatology, with clinical manifestations debuting at an advanced age. Variant p.Del Glu23_Lys24, despite of presenting a mild symptomatology, its phenotype has developed at an early age (28 years), so it is convenient to follow up this patient in order to study the evolution of the symptomatology. The p.His379Asn variant is associated with a phenotype from moderate to severe of HPP probably childhood onset without bone impairment. The drastic decrease of the ALP activity could be explained mainly by the loss of Zn-binding capacity of TNSALP. The treatment of the patient affected with this variant with asfotase alpha therapy has shown an important improvement of the clinical manifestations suggesting that the main symptoms of this patient are related to HPP disease.

Supporting our results, the VarSome web has reclassified the study variants as likely pathogenic (p.Del Glu23_Lys24) and pathogenic (p.Pro292Leu; p.His379Asn).

The characterization and subsequent classification of new *ALPL* gene mutations found in HPP patients may facilitate disease management by healthcare professionals. This allows the disease to be addressed avoiding misdiagnosis or/and mistreatment and improving the quality of life of patients.

## Data Availability Statement

The datasets presented in this article are not readily available because they have been generated by a hospital service under a privacy clause. Requests for access to the datasets should be addressed to the corresponding author Cristina Garcia-Fontana.

## Ethics Statement

The studies involving human participants were reviewed and approved by the ethics committee of the University Hospital San Cecilio of Granada in accordance with the principles of the World Medical Association Declaration of Helsinki (Project ID: 0777-M1-20. Research Ethics Committee of Granada Center (CEI-Granada) on 8 May 2019). The patients/participants provided their written informed consent to participate in this study.

## Author Contributions

Study design: MM-T, BG-F,VC-B, RS-dT, CG-F, JMV-S, FA-V, SG-S, II-B, and LM-H. Study conduct: JV-S, CG-F, FA-V, BG-F, SG-S, GM-N, PR, MC-S, II-B, RS-dT, and LM-H. Data collection: JMV-S, CG-F, BG-F, FA-V, and SG-S. Data analysis: JMV-S, CG-F, BG-F, FA-V, SG-S, II-B, RS-dT, and VC-B. Data interpretation: FA-V, RS-dT, LM-H, SG-S, JMV-S, CG-F, BG-F, and MM-T. Drafting manuscript: RS-dT, BG-F, CG-F, FA-V, MM-T, and II-B. Revising manuscript: MM-T, BG-F, CG-F, II-B, and RS-dT. All authors contributed to the article and approved the submitted version.

## Funding

This research was funded by the Instituto de Salud Carlos III grants (PI18-00803, PI21/01069 and PI18-01235), co-funded by the European Regional Development Fund (FEDER) and by Junta de Andalucía grant (PI-0268-2019). In addition, VC-B is supported by postdoctoral fellowship from Junta de Andalucía (RH-0141-2020) and JMV-S and SG-S are funded by predoctoral fellowships from Instituto de Salud Carlos III (CM19/00188 and FI19/00118 respectively). CG-F and RS-dT are funded by postdoctoral Sara Borrell fellowship and Research investigator grant in the framework of the youth guarantee Program from the Instituto de Salud Carlos III and the University of Granada with co-funding by FEDER respectively (CD20/00022 and 8110 grant number). GM-N is supported by the predoctoral program from Instituto de Salud Carlos III (FI17/00178) and PR is a Ramon y Cajal Researcher from the MINECO (RYC-2015-18383) at GENyO and University of Granada. The funders had no role in the design of the study; in the collection, analyses, or interpretation of data; in the writing of the manuscript, or in the decision to publish the results.

## Conflict of Interest

The authors declare that the research was conducted in the absence of any commercial or financial relationships that could be construed as a potential conflict of interest.

## Publisher’s Note

All claims expressed in this article are solely those of the authors and do not necessarily represent those of their affiliated organizations, or those of the publisher, the editors and the reviewers. Any product that may be evaluated in this article, or claim that may be made by its manufacturer, is not guaranteed or endorsed by the publisher.
